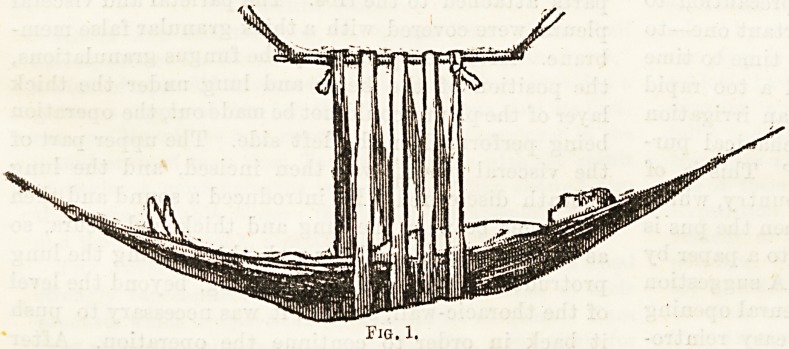# Bone, Joint, and Orthopædic Surgery

**Published:** 1894-09-08

**Authors:** 


### PROGRESS IN SURGERY.

### BONE, JOINT, AND ORTHOPÆDIC SURGERY.

#### Orthopædic Surgery.

**Pott's Disease—Recumbency in the Treatment of.**—Schapps[10] advocates the use of a bed on rubber-tyred wheels, the frame of which is made of gas-pipe and the body of canvas, stretched as tightly as possible, in which a hole is cut at the point where the rectum is placed; pads are sewn on to prevent undue pressure on bony points and to steady the body; straps are also fixed to the bed, which pass over the shoulders from the axillæ and buckle to the bed as near the root of the neck as possible. By means of a weight attached to the ordinary Sayre headpiece, traction is made on the spine, and in many cases supplemented by counter-traction applied to a pelvic band. Keen,[11] in a paper read before the Orthopædic Section of the College of

Physicians of Philadelphia, gives an excellent *resumé* of the surgical treatment of Pott's disease, especially dwelling on aggressive procedures for abscess, and discussing the value of laminectomy.

Phelps,[12] in a paper on Pott's disease, pointed out that cases of this disease are now properly classed under several sub-divisions—viz., a tubercular form, slow in development; a form due to acute osteomyelitis, in which the onset is sudden; a condition occurring in the course of some fevers, especially typhoid, and closely resembling Pott's disease; also that due to actinomycosis. To these might be added the cases of angular deformity due to syphilis and cancer.

**Plaster Jackets.**—Gehrung,[13] points out the necessity, in cases of cervical spinal disease, of fixing the "jury-mast," or other apparatus, to a jacket which reaches below the crests of the ilia, and not merely rests on the shoulders. Sloan[14] advocates its application in the horizontal position, the patient lying in a hammock, the sagging of the spine being prevented by three or four turns of a bandage passing round the body outside the hammock to a horizontal bar above (Fig. 1).

**Flat-Foot.—Operative Treatment of.**—Gleich,[15] on the ground that he has seen by all other methods relapse and recurrence of symptoms, proceeds as follows. An incision is made similar to that for Pirogoff's amputation, followed by a tenotomy of the tendo Achillis. From the calcaneus is then resected in a diagonal plane from above downwards and behind forwards a wedge having its base downwards and measuring about half an inch in its thickest part. If the remaining cut surfaces of the calcaneus are now applied to each other, it will be seen that the angle has been decreased between the axis of the bone and the plantar plane, and that the foot has been raised about three-eighths of an inch.

FIG. 1.

**Club-Foot—Operative Treatment of.**—Hartley[16] reports twenty-five cases which show the great value of operation where mechanical means have failed. Whilst not believing in any routine operative treatment of club-foot, he holds that, "in the first and second degrees of deformity, when treatment is begun shortly after birth, traction, stretching, massage, and proper fixation generally suffice. When these are found insufficient, tenotomy of the tendo Achillis, tibialis posticus, or Phelps' operation, with or without fasciotomy: or, in cases of the paralytic variety, arthrodesis of the ankle-joint, may be indicated. In the congenital club-foot of this variety complete extirpation of the astragalus is recommended. In the acquired variety of paralytic origin a cuneiform osteotomy, including the prominent angle of the astragalus and adjoining surface of the tibia is indicated, as it corrects the deformity and produces a syndesmosis between the astragalus and the tibia. In inveterate cases, where the ankle cannot be flexed to a right angle, and the supination of the calcaneus is marked, the extirpation of the astragalus and the division of the calcaneo-fibular ligament is necessary, in addition to cuneiform osteotomy of the tarsus. Unless the equinus is completely removed, and the supination in the calcaneus is corrected, every step made by the patient aids a return of his varus. It is imperative, if we wish to obtain a permanent and good result, to relieve all deformity completely and at once. This ought to be accomplished before the weight of the body is allowed to act upon the foot. The clearest evidence of an imperfect operation is the need of an apparatus and support after correction has been accomplished." With the latter remark we fail to agree.

**Rickets.**—Subluxation of Clavicle.—Gibney[17] remarks: "I find a number of cases of lateral curvature in rhachitic subjects who have a displacement of the external end of the clavicle, and it is aggravated by certain exercises used for the correction of the curvature. Latterly, we have a method of curing these that is very good, namely, the injection of alcohol around the articulation, binding the parts with a roller bandage. Two or three injections set up inflammation round the joint."

**Osteotomy for Rhachitic Deformity.**—In the *Clinical Journal*[18] H. H. Clutton gives a clear *resumé* of the various proceedings in vogue and the indications for their use, especially when division at and near the epiphysis is necessary, or when it is better to divide the shaft itself.

**Dupuytren's Contraction.**—Abbé[19], from a consideration of forty cases, discards Adams' and Keen's theory of the gouty origin of this deformity. He believes it due to traumatism of the nerve ends in the palm, setting up reflex contraction. Cases are quoted in support of this theory, but we venture to think that the truth lies in a mean of these opinions. Persons with a gouty inheritance are particularly prone to Dupuytren's contraction, if the palm be injured. Abbé prefers the open incision into the palm and complete excision of the affected fascia.

**Mal-Development.**—A. H. Tubby contributes to the *Lancet*[20] a case of "Lobster-claw deformity of the feet and partial suppression of the fingers with remarkable hereditary history." The patient, a boy aged 3, was one of a family, all of whom presented abnormalities of the hands and feet. In his case the feet gave one the idea of a lobster's claw. The second, third, and fourth toes were entirely suppressed, the great toe was much lengthened, and the fifth toe was also overgrown. Between these two was a wide sulcus. But the most remarkable point was that the great toe presented, in addition to the ordinary movements of flexion and extension, the power of opposing itself to the remaining toe, so that in its action and grasping movements it resembled the thumb of man and the opposing toe of the quadrumana. In the case of a younger child, a girl, the feet presented the same appearance, while in place of there being one finger on each hand, there were two. The deformity had persisted nearly constantly through four generations.

[10] N. Y. Med. Jour., Oct. 21st, 1893. [11] Therap. Gaz., Jan. 15th, 1894. [12] N. Y. Med. Record, March 17th, 1894, p. 343. [13] Philadelphia Med. News, Sept. 9th, 1893. [14] Med. Rec., March 10th, 1894, p. 318. [15] Amer. Jour. Med. Science, Feb. 1894, p. 209. [16] Annals of Surg., March, 1894. [17] Internat. 1894., vol. iv., 1894. [18] Feb. 14th, 1894. [19] N. Y. Med. Jour,, Jan. 13th, Clin. [20] Feb. 17th, 1894.

## Figures and Tables

**Fig. 1. f1:**